# Supplementary material for: Towards unified AI-driven fracture mechanics: the extended deep energy method (XDEM)
Source: Nat Commun. 2026 Aug 15;17:8492. doi: 10.1038/s41467-026-76748-1 (PMC13484471; doi:10.1038/s41467-026-76748-1)
Supplement: Supplementary file 3 [file 41467_2026_76748_MOESM3_ESM.zip › pic/Method/williams/crack_u2_displacement_left_T.pdf]

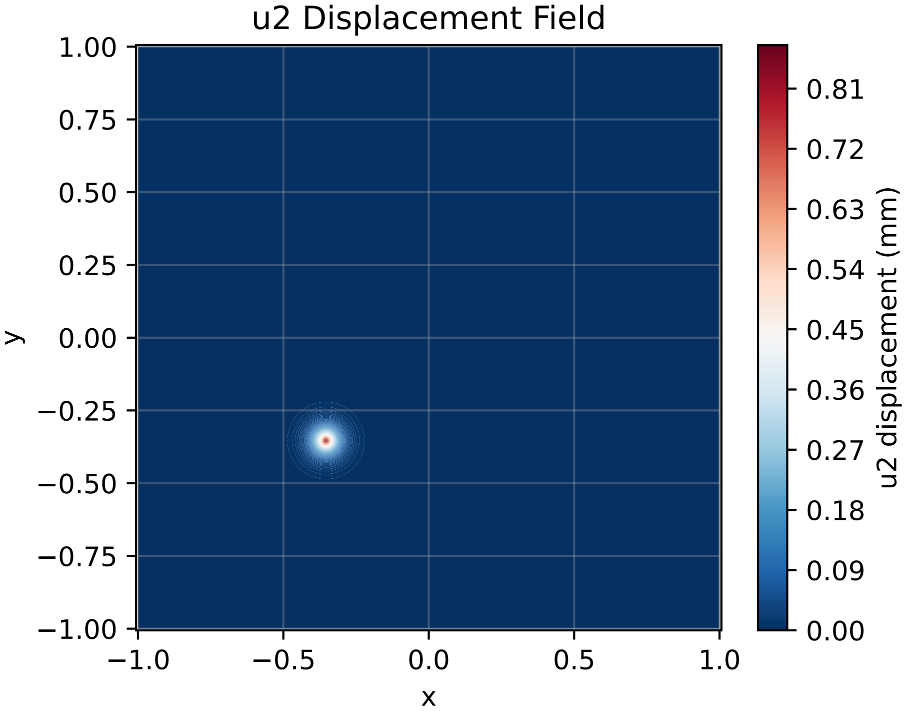

u2 Displacement Field
1.00
0.75
0.50
0.25
0.00
−0.25
−0.50
−0.75
−1.00
y
−1.0
−0.5
0.0
0.5
1.0
x
0.81
0.72
0.63
0.54
0.45
0.36
0.27
0.18
0.09
0.00
u2 displacement (mm)
